# Racial and Ethnic Differences in Potentially Inappropriate Medication Use Among Medicare Beneficiaries

**DOI:** 10.1001/jamanetworkopen.2025.4763

**Published:** 2025-04-14

**Authors:** Eli Raver, Jeah Jung, Caroline Carlin, Roger Feldman, Sheldon Retchin, Wendy Xu

**Affiliations:** 1 Division of Health Services Management and Policy, College of Public Health, The Ohio State University, Columbus; 2 Department of Health Administration and Policy, College of Public Health, George Mason University, Fairfax, Virginia; 3 Department of Family Medicine and Community Health, School of Medicine, University of Minnesota Twin Cities, Minneapolis; 4 Division of Health Policy and Management, School of Public Health, University of Minnesota Twin Cities, Minneapolis; 5 Division of General Internal Medicine, College of Medicine, The Ohio State University, Columbus

## Abstract

**Importance:**

Older Medicare beneficiaries are susceptible to receiving potentially inappropriate medications (PIMs), where the risks outweigh the benefits. Racial and ethnic differences in PIM use may perpetuate health disparities and disproportionately lead to costly adverse drug events for some groups.

**Objective:**

To examine associations of race and ethnicity with PIM use among older Medicare beneficiaries.

**Design, Setting, and Participants:**

This cross-sectional study used nationwide Medicare Part D Event files, fee-for-service claims, and Medicare Advantage (MA) encounter data among Medicare beneficiaries 65 years or older with Part D prescription drug coverage. The study population included random samples of 20% of traditional Medicare (TM) beneficiaries and 50% of MA enrollees from January 1, 2016, to December 31, 2019. Analysis was conducted from May 28 to September 16, 2024.

**Exposure:**

Race and ethnicity.

**Main Outcomes and Measures:**

Linear probability models were estimated for outcomes of (1) high-risk medication use among older adults, (2) potentially harmful drug-disease interactions in patients with dementia, and (3) potentially harmful drug-disease interactions in patients with a history of falls.

**Results:**

The study sample included 21 193 170 patients with 32 199 587 beneficiary-year observations (mean [SD] age, 75.5 [7.3] years; 18 936 697 [58.8%] female; 983 513 [3.1%] Asian or Pacific Islander, 3 190 515 [9.9%] Black, 2 710 930 [8.4%] Hispanic, and 25 314 629 [78.6%] White). Compared with White beneficiaries, the rates of high-risk medication use were 1.7 [95% CI, −1.8 to −1.6] percentage points lower for Asian or Pacific Islander beneficiaries, 3.4 [95% CI, −3.4 to −3.3] percentage points lower for Black beneficiaries, and 0.6 [95% CI, −0.6 to −0.5] percentage points lower for Hispanic beneficiaries. Similarly, White beneficiaries had the highest rates of potentially harmful drug-disease interactions among those with dementia as well as those with a history of falls. The pattern of racial and ethnic differences was similar in analyses stratified by enrollment in TM or MA plans. However, the differences between White individuals and other groups were smaller in MA than in TM plans for all comparisons.

**Conclusions and Relevance:**

In this cross-sectional study of 32 199 587 Medicare beneficiary-years from 2016 to 2019, White Medicare beneficiaries had consistently higher rates of PIM use compared with other racial and ethnic groups. The observed differences may be partially explained by racial and ethnic differences in overall prescription drug use and suggest the need to reduce PIM use in all racial and ethnic groups.

## Key Points

**Question:**

Are there racial and ethnic differences in the use of potentially inappropriate medications (PIMs) among older Medicare beneficiaries?

**Findings:**

In this cross-sectional study of more than 32 million Medicare beneficiary-years from 2016 to 2019, White beneficiaries had consistently higher rates of PIM use compared with Asian or Pacific Islander, Black, and Hispanic beneficiaries.

**Meaning:**

These findings suggest the need to reduce PIM use among all racial and ethnic groups, particularly White Medicare beneficiaries.

## Teaser

This cross-sectional study examines differences in potentially inappropriate medication use by race and ethnicity among Medicare Advantage and traditional Medicare beneficiaries and compares the racial and ethnic differences between insurance coverage types.

## Introduction

Potentially inappropriate medications (PIMs) have a greater risk of harm than benefit for older adults,^1^ yet they remain widely used.^2,3,4^ The pharmacology of many PIMs changes with older age, leading to reduced metabolism and clearance from the body, increased sensitivity to adverse effects, or reduced therapeutic effect.^1^ While some medications should be generally avoided in all older adults, others are considered inappropriate because they interact with specific health conditions by worsening symptoms or impeding treatment. PIMs are associated with an increased risk of adverse drug events,^5^ falls,^6^ hospitalizations,^7^ and higher health care costs.^8,9^

While previous studies have illustrated differences in PIM use by racial and ethnic groups, findings have been inconsistent across patient populations and clinical situations. For example, older White adults appear to have higher PIM use generally,^10,11^ but older Black adults may have greater use of medications that increase fall risk,^12^ such as benzodiazepines and anticholinergic agents,^10^ and there may be minimal racial and ethnic differences in PIM use that can exacerbate dementia.^13^ If racial and ethnic minority groups receive disproportionately more PIMs, this may perpetuate health disparities, particularly for socioeconomically disadvantaged groups who bear the cost of adverse drug events. For these reasons, assessing patterns of PIM use among different racial and ethnic groups is an important step to ensure equitable quality of care.^14^

The past decade has witnessed changes in Medicare with the rapid growth in Medicare Advantage (MA) enrollment. MA plans now cover more than half of Medicare beneficiaries.^15^ Because of this recent shift to MA enrollment, most prior studies of PIM use were conducted at a time when Medicare was predominantly fee-for-service.^16^ Although it offered valuable insights into racial and ethnic differences in PIM use among Medicare beneficiaries, 1 recent study was limited to MA data from a single pharmacy benefit manager.^10^ Because MA enrollment growth has been disproportionately faster among Asian, Black, Hispanic, and Pacific Islander beneficiaries,^17^ it is important to address the literature gap by examining PIM use differences by race and ethnicity in MA and traditional Medicare (TM), as well as by comparing the racial and ethnic differences between insurance coverage types.

Medicare requires that standalone Part D prescription drug plans and MA plans publicly report information about PIM use among their enrollees. Although separate reporting by racial and ethnic group is not required, the Centers for Medicare & Medicaid Services (CMS) will implement a health equity index to adjust star rating calculations for Part D quality measures, starting in 2027.^18^ Thus, it will be necessary for payers and patients to better understand how quality of care differs across racial and ethnic groups according to enrollment choices. Therefore, we examined the current associations of race and ethnicity with PIM use among older Medicare beneficiaries.

## Methods

### Data

This cross-sectional study used Part D Event Files from January 1, 2016, to December 31, 2019, which provide outpatient prescription drug claims, to construct measures of PIM use. These files include details on drug name, strength, days’ supply, and prescription fill date. We used TM claims and MA encounter data to assess eligibility criteria for PIM measures. Claims and encounter data were also used to construct health risk scores based on diagnoses in prior-year claims (or encounters) for inpatient and outpatient services. We used the Master Beneficiary Summary File for beneficiary demographics, zip code and county of residence, and monthly Medicare and Medicaid enrollment details, including plan type. We also linked county health care resources from the Area Health Resource File and zip code socioeconomic characteristics from the American Community Survey. The Ohio State University institutional review board exempted this study from review and waived the informed consent requirement for this retrospective study using deidentified secondary data. This study followed the Strengthening the Reporting of Observational Studies in Epidemiology (STROBE) guidelines for reporting cross-sectional studies.

### Study Sample

We identified Medicare beneficiaries 65 years or older with continuous Medicare Part A, B, and D coverage for 12 months, excluding those who switched between TM and MA coverage midyear. Due to concerns of missing records in MA encounter data, we followed established and validated methods to limit the analyses to contracts with highly complete data.^19,20^ We randomly selected 50% of the enrollees from MA plans from contracts with highly complete encounter data to reduce the computational burden. We used a random 20% sample of TM beneficiaries with the same distribution of residence by US state as the MA enrollees for each year. We excluded beneficiaries who were eligible for Medicare solely due to end-stage kidney disease, as they were enrolled only in TM during the study period and may have substantially different patterns of health care service use than other Medicare beneficiaries.^21^ We also excluded those living outside the 50 US states or Washington, DC. A sample flow diagram is provided in the eFigure in Supplement 1.

### Exposures

The key explanatory variable was beneficiary race and ethnicity based on the Research Triangle Institute (RTI) race code included in Master Beneficiary Summary File data. Compared with the race code from the Medicare enrollment database, the RTI race code improves accuracy of race and ethnicity classification, particularly for Asian, Hispanic, and Pacific Islander individuals.^22,23^ We included beneficiaries whose race and ethnicity was coded as Asian or Pacific Islander, Black, Hispanic, or White. Following the RTI race code,^23^ Hispanic individuals of any race were categorized as Hispanic, while all other groups were of non-Hispanic ethnicity. Similarly, Asian and Pacific Islander individuals were grouped together. We excluded individuals whose race was coded as American Indian or Alaska Native, other, or unknown, as these groups cumulatively represented less than 1% of the sample and were too small for data analyses.

### Outcomes

We measured 3 PIM outcomes, following definitions from the Healthcare Effectiveness Data and Information Set (HEDIS) quality measures.^2,3,24^ These PIM measures represent potentially harmful medication use among older adults, especially for select populations with high care needs or high costs of care, where there is clear guidance for avoiding these medications.^1,25,26^

The first outcome was the use of high-risk medications in Medicare beneficiaries 65 years or older. This measure included events when the same high-risk medication was dispensed at least twice during a calendar year. These medications, such as anticholinergics, long-acting sulfonylureas, and skeletal muscle relaxants, incur risks that outweigh the benefits for older patients because the adverse effects become more severe or the therapeutic benefits decrease as patients age.^27^

The second outcome was a potentially harmful drug-disease interaction in patients with dementia. This measure captured dispensing events for drug classes, such as antipsychotics or benzodiazepines, that have been shown to exacerbate dementia through impaired cognition, sleep disturbances, and uncoordinated movement. We identified individuals with dementia following the HEDIS algorithms, based on claims or encounters associated with a diagnosis of dementia, or a filled prescription for a medication used to treat dementia.^24^

The third outcome was a potentially harmful drug-disease interaction in patients who had a recent accidental fall or hip fracture. This measure captured dispensing events for drug classes, such as sedative hypnotics and tricyclic antidepressants, that can increase the risk of falls in older adults. Accidental falls and fractures were identified by inpatient, outpatient, observation, or emergency department visits associated with a diagnosis of an accidental fall or hip fracture occurring before the PIM event, in the same calendar year or previous year.

The drug classes of potentially inappropriate medications and excluded conditions for each outcome are detailed in the eMethods in Supplement 1. We created separate analytic samples for each outcome following the eligibility criteria.

### Statistical Analysis

We estimated linear probability models for the differences in rates of PIM use by race and ethnicity. Because individuals could be observed multiple times during the study period, we used robust SEs clustered by individual enrollees. We performed primary analyses that pooled TM beneficiaries and MA enrollees together to assess the overall racial and ethnic differences in PIM use. We further performed stratified analyses for individuals in TM and MA plans to allow for the possibility of different patterns in racial and ethnic PIM use across the 2 samples. We used a generalized Hausman test to compare race and ethnicity differences between the TM and MA strata.^28^

Individual control variables for the primary analyses included age, sex, MA enrollment, Medicaid-Medicare dual eligibility, the claims-based frailty index of Kim et al,^29^ and health status measured by the Hierarchical Condition Category risk score. We calculated risk scores without using diagnoses from health risk assessment and chart review to adjust for high coding intensity for MA enrollees.^30^ Zip code–level characteristics included rurality, percentage of adults with a college degree, median household income, and percentage of the population speaking only English. Time-varying county health resources included densities of hospital beds and physicians. County fixed effects controlled for time-invariant county characteristics, and year fixed effects accounted for secular trends. The control variables for the stratified analyses were the same as for the primary analyses, except that the samples were stratified by MA enrollment. Analyses were conducted from May 28 to September 16, 2024. Two-sided *P* < .05 indicated statistical significance.

## Results

As shown in Table 1, there were 21 193 170 patients eligible for the outcome of high-risk medication use in older adults, totaling 32 199 587 beneficiary-year observations. Of these, 983 513 (3.1%) were coded as Asian or Pacific Islander, 3 190 515 (9.9%) as Black, 2 710 930 (8.4%) as Hispanic, and 25 314 629 (78.6%) as White. The mean (SD) age was 75.5 (7.3) years; 18 936 697 (58.8%) were female and 13 262 890 (41.2%) were male; and 19 420 301 (60.3%) were enrolled in MA plans. Sample characteristics for the other outcomes were similar (eTables 1 and 2 in Supplement 1).

**Table 1.** Characteristics of Sample Eligible for Measure of High-Risk Medication Use in Older Adults

Characteristic	Racial and ethnic group^a^				
	Overall	Asian or Pacific Islander	Black	Hispanic	White
No. of beneficiary-year observations	32 199 587	983 513	3 190 515	2 710 930	25 314 629
No. of patients	21 193 170	674 593	2 060 148	1 757 242	16 701 187
Age, mean (SD), y	75.5 (7.3)	75.1 (7.2)	74.8 (7.1)	75.1 (7.1)	75.7 (7.3)
Sex, No. (%)					
Female	18 936 697 (58.8)	552 296 (56.2)	2 027 564 (63.5)	1 567 294 (57.8)	14 789 543 (58.4)
Male	13 262 890 (41.2)	431 217 (43.8)	1 162 951 (36.5)	1 143 636 (42.2)	10 525 086 (41.6)
Rural residence, No. (%)	6 419 873 (19.9)	35 647 (3.6)	435 278 (13.6)	249 549 (9.2)	5 699 489 (22.5)
Dual eligibility, No. (%)	4 874 411 (15.1)	366 901 (37.3)	985 741 (30.9)	1 131 022 (41.7)	2 390 747 (9.4)
Medicare Advantage enrollment, No. (%)	19 420 301 (60.3)	622 192 (63.3)	2 329 201 (73.0)	1 986 706 (73.3)	14 482 202 (57.2)
HCC risk score, mean (SD)^b^	1.10 (1.00)	0.97 (0.88)	1.26 (1.16)	1.24 (1.08)	1.07 (0.97)
Claims-based frailty index, mean (SD)^c^	0.16 (0.06)	0.15 (0.06)	0.17 (0.07)	0.17 (0.07)	0.16 (0.06)
Zip code median household income, mean (SD), $ US	65 610 (25 815)	80 275 (31 387)	51 696 (21 508)	57 973 (22 892)	67 611 (25 533)
Residents in zip code with a 4-y degree, mean (SD), %	31.0 (16.4)	39.0 (17.5)	25.2 (14.3)	25.2 (14.8)	32.1 (16.4)
Zip code households speaking only English, mean (SD), %	81.6 (19.0)	62.0 (21.7)	82.2 (17.4)	51.2 (26.1)	85.6 (14.2)
No. of hospital beds per 1000 population (county level), mean (SD)	3.00 (2.01)	2.81 (1.34)	3.68 (2.40)	2.97 (1.38)	2.92 (2.02)
No. of physicians per 1000 population (county level), mean (SD)	3.31 (2.27)	4.32 (2.33)	4.00 (2.57)	3.55 (2.18)	3.16 (2.21)

Abbreviation: HCC, Hierarchical Condition Category.

^a^ Percentages are calculated using the number of beneficiary-year observations as the denominator.

^b^ Observed scores range from 0 to 20.6, with higher scores indicating higher predicted health care expenditures.

^c^ Scores range from 0 to 1, with higher scores indicating greater frailty.

Table 2 shows the unadjusted rates of PIM use for each outcome by racial and ethnic group. Overall, high-risk medication use among older adults was prevalent for 107 400 beneficiary-years (10.9%) among Asian or Pacific Islander adults, 402 696 (12.6%) among Black adults, 373 203 (13.8%) among Hispanic adults, and 3 591 313 (14.2%) among White adults. Potentially harmful drug-disease interactions in older adults with dementia were prevalent for 20 158 beneficiary-years (27.7%) among Asian or Pacific Islander adults, 73 262 (25.3%) among Black adults, 96 649 (36.7%) among Hispanic adults, and 614 357 (33.5%) among White adults. Potentially harmful drug-disease interactions in older adults with a history of falls were prevalent for 8241 beneficiary-years (22.6%) among Asian or Pacific Islander adults, 34 400 (26.7%) among Black adults, 34 130 (29.3%) among Hispanic adults, and 401 132 (31.2%) among White adults. The rates of PIM use by race and ethnicity for the stratified samples of TM beneficiaries and MA enrollees followed the same pattern as for the pooled sample. For any given measure and racial or ethnic group, TM beneficiaries had higher unadjusted rates and MA enrollees had lower unadjusted rates compared with the total sample.

**Table 2.** Unadjusted Rates of Potentially Inappropriate Medication Use by Race, Ethnicity, and Enrollment Status

Enrollment status	Asian or Pacific Islander	Black	Hispanic	White
**High-risk medication use among older adults, No./total No. (%)**				
Overall sample (N = 32 199 587)	107 400/983 513 (10.9)	402 696/3 190 515 (12.6)	373 203/2 710 930 (13.8)	3 591 313/25 314 629 (14.2)
Traditional Medicare (n = 12 779 286)	45 786/361 321 (12.7)	121 717/861 314 (14.1)	112 654/724 224 (15.6)	1 707 825/10 832 427 (15.8)
Medicare Advantage (n = 19 420 301)	61 614/622 192 (9.9)	280 979/2 329 201 (12.1)	260 549/1 986 706 (13.1)	1 883 488/14 482 202 (13.0)
**Potentially harmful drug-disease interactions in older adults with dementia, No./total No. (%)**				
Overall sample (N = 2 459 218)	20 158/72 835 (27.7)	73 262/289 232 (25.3)	96 649/263 494 (36.7)	614 357/1 833 657 (33.5)
Traditional Medicare (n = 1 053 606)	10 361/34 084 (30.4)	26 274/95 107 (27.6)	33 469/84 269 (39.7)	300 730/840 146 (35.8)
Medicare Advantage (n = 1 405 612)	9797/38 751 (25.3)	46 988/194 125 (24.2)	63 180/179 225 (35.3)	313 627/993 511 (31.6)
**Potentially harmful drug-disease interactions in older adults with a history of falls, No./total No. (%)**				
Overall sample (N = 1 569 221)	8241/36 432 (22.6)	34 400/129 059 (26.7)	34 130/116 344 (29.3)	401 132/1 287 386 (31.2)
Traditional Medicare (n = 675 608)	4135/15 647 (26.4)	11 426/37 434 (30.5)	11 875/34 351 (34.6)	205 547/588 176 (34.9)
Medicare Advantage (n = 893 613)	4106/20 785 (19.8)	22 974/91 625 (25.1)	22 255/81 993 (27.1)	195 585/699 210 (28.0)

Regression results (Table 3) indicate that White beneficiaries had the highest rates of PIM use. Compared with White beneficiaries (reference group), the rates of high-risk medication use were 1.7 (95% CI, −1.8 to −1.6) percentage points lower for Asian or Pacific Islander beneficiaries, 3.4 (95% CI, −3.4 to −3.3) percentage points lower for Black beneficiaries, and 0.6 (95% CI, −0.6 to −0.5) percentage points lower for Hispanic beneficiaries. Similarly, the rates of potentially inappropriate drug-disease interactions in older adults with dementia were 3.8 (95% CI, −4.3 to −3.4) percentage points lower for Asian or Pacific Islander beneficiaries, 11.2 (95% CI, −11.5 to −11.0) percentage points lower for Black beneficiaries, and 0.5 (95% CI, −0.8 to −0.3) percentage points lower for Hispanic beneficiaries, compared with White beneficiaries. The rates of potentially inappropriate drug-disease interactions in older adults with a history of falls were 6.5 (95% CI, −7.0 to −6.0) percentage points lower for Asian or Pacific Islander beneficiaries, 8.3 (95% CI, −8.6 to −8.0) percentage points lower for Black beneficiaries, and 2.1 (95% CI, −2.4 to −1.7) percentage points lower for Hispanic beneficiaries compared with White beneficiaries.

**Table 3.** Association of Race and Ethnicity With Potentially Inappropriate Medication Use, by Medicare Type

Race or ethnicity	Percentage point differences (95% CI)^a^		
	Overall sample^b^	TM beneficiaries^c^	MA enrollees^c^
**High-risk medication use among older adults**			
Asian or Pacific Islander	−1.7 (−1.8 to −1.6)	−2.0 (−2.1 to −1.9)	−1.4 (−1.5 to −1.3)
Black	−3.4 (−3.4 to −3.3)	−4.3 (−4.4 to −4.2)	−2.9 (−2.9 to −2.8)
Hispanic	−0.6 (−0.6 to −0.5)	−1.3 (−1.5 to −1.2)	−0.1 (−0.1 to 0.01)
**Potentially harmful drug-disease interactions in older adults with dementia**			
Asian or Pacific Islander	−3.8 (−4.3 to −3.4)	−5.3 (−5.9 to −4.7)	−2.7 (−3.4 to −2.1)
Black	−11.2 (−11.5 to −11.0)	−12.8 (−13.2 to −12.4)	−10.2 (−10.5 to −9.9)
Hispanic	−0.5 (−0.8 to −0.3)	−1.6 (−2.1 to −1.1)	0.5 (0.1 to 0.9)
**Potentially harmful drug-disease interactions in older adults with a history of falls**			
Asian or Pacific Islander	−6.5 (−7.0 to −6.0)	−7.7 (−8.5 to −6.9)	−5.6 (−6.2 to −4.9)
Black	−8.3 (−8.6 to −8.0)	−10.5 (−11.0 to −9.9)	−7.1 (−7.4 to −6.7)
Hispanic	−2.1 (−2.4 to −1.7)	−3.0 (−3.7 to −2.4)	−1.4 (−1.9 to −1.0)

Abbreviations: MA, Medicare Advantage; TM, traditional Medicare.

^a^ Represents the mean difference in outcomes between a given racial or ethnic group and the White group (reference group), based on regression models. For each percentage point difference reported, the difference between estimates for TM beneficiaries and MA enrollees was statistically significant (*P* < .001), based on a generalized Hausman test.

^b^ The regression models for the overall sample controlled for Medicare type (TM vs MA), individual characteristics, zip code socioeconomic characteristics, county health resource availability, and fixed effects for year and county.

^c^ The stratified regression models for TM beneficiaries and MA enrollees controlled for individual characteristics, zip code socioeconomic characteristics, county health resource availability, and fixed effects for year and county.

Stratified regression results for TM beneficiaries and MA enrollees (Table 3) followed a similar pattern to the pooled results. The generalized Hausman tests showed that racial and ethnic differences were significantly larger among TM beneficiaries compared with the differences among MA enrollees. For example, Black beneficiaries in TM had a rate of high-risk medication use that was 4.3 (95% CI, −4.4 to −4.2) percentage points lower compared with White beneficiaries in TM, whereas Black enrollees in MA plans had a rate 2.9 (95% CI, −2.9 to −2.8) percentage points lower, compared with White enrollees in MA plans (*P* < .001). Similarly, Asian or Pacific Islander beneficiaries in TM had a rate of high-risk medication use that was 2.0 (95% CI, −2.1 to −1.9) percentage points lower compared with White beneficiaries in TM, whereas Asian or Pacific Islander enrollees in MA plans had a rate 1.4 (95% CI, −1.5 to −1.3) percentage points lower compared with White enrollees in MA plans (*P* < .001). However, there was no significant difference in high-risk medication use between White and Hispanic MA enrollees (−0.1 [95% CI, −0.1 to 0.01] percentage points). Hispanic enrollees with dementia in MA plans had a rate of potentially harmful drug-disease interactions that was 0.5 (95% CI, 0.1-0.9) percentage points higher compared with White enrollees.

## Discussion

In this cross-sectional study of racial and ethnic differences in PIM use among older Medicare beneficiaries, we found that White beneficiaries consistently had the highest rates of PIM use, followed by Hispanic and Asian or Pacific Islander beneficiaries. Black beneficiaries consistently had the lowest rates. This pattern was consistent for all PIM measures and for both TM beneficiaries and MA enrollees. Higher PIM use among White Medicare beneficiaries puts them at an increased risk of costly adverse drug events compared with other racial and ethnic groups.^9^

Our findings are mostly consistent with an earlier study by Figueroa et al^11^ noting that White individuals used more high-risk medications than other groups. While we observed that Black individuals had the lowest PIM use, Figueroa et al^11^ observed that Black individuals used more high-risk medications than Asian or Pacific Islander and Hispanic individuals. Although we found that White Medicare beneficiaries had higher PIM use overall, previous studies limited to specific conditions or clinical settings have produced inconsistent findings. For example, older Hispanic adults have higher inappropriate use of psychotropic medications^31^ and higher PIM use among patients with heart failure or multiple chronic conditions.^32,33^ On the other hand, older White adults have higher reported rates of inappropriate use of antibiotics in the emergency department^34^ and higher PIM use among patients with Parkinson disease.^35^ We also found that White Medicare beneficiaries were more likely to receive drugs that can worsen dementia or increase the risk of falls.

One possible explanation for our finding of lower PIM use among racial and ethnic minority groups is that the outcome measures reward lower rates of prescription drug use. Thus, the racial and ethnic differences in PIM use that we observed may be partially explained by racial and ethnic differences in overall use of prescription drugs, including medications that are beneficial.^16,36^ Other studies have shown that older White adults have the highest rates of prescription drug use, with older Black and Hispanic adults using fewer prescription drugs.^37,38,39,40^ This may have contributed to a relatively higher risk for PIM use among White individuals. Lower use of prescription drugs among racial and ethnic minority groups may result from multiple factors, such as limited access to primary care,^41^ specialists,^42^ and pharmacies^40^; greater sensitivity to costs^43,44^; clinician bias^45^; or an aversion to prescription medications based on cultural preferences or distrust of the health care system.^46^

The overall patterns of racial and ethnic differences in PIM use were reflected in separate analyses of TM beneficiaries and MA enrollees. However, we found that the racial and ethnic differences were smaller in MA plans compared with TM. MA plans often implement utilization management practices such as prior authorization, and they selectively contract with high-quality health care professionals to promote low-cost, high-quality care.^47,48,49^ The standardization of care quality and lower out-of-pocket prescription drug costs in MA plans may reduce racial and ethnic differences in PIM use among MA enrollees.^50,51^ It is also possible that the quality of selected MA contracts with highly complete data is better than average. The smaller racial and ethnic differences in PIM use that we observed among MA enrollees were likely not due to differences in access to care between TM and MA plans, as previous studies have shown similar racial and ethnic gaps in access to care, including prescription medications, across TM and MA plans.^52,53^

Our findings demonstrate that racial and ethnic differences in quality of medication therapy should be monitored in both TM and MA plans. Furthermore, future monitoring should distinguish between overutilization of PIMs and underutilization of appropriate medications, while accounting for differences in access to care. CMS should use recent PIM use patterns by race and ethnicity to strategically focus its health equity initiatives. Future studies should evaluate whether implementing the health equity index in CMS star ratings reduces racial and ethnic differences in Medicare quality measures, including PIM use.^18^

### Limitations

This study has several limitations. First, the sample sizes were not large enough to include American Indian and Alaska Native individuals, who have particular health care needs and varied health care access through the Indian Health Service.^54^ Second, the race and ethnicity classification system available in the data did not allow more granular examination of heterogeneous racial and ethnic subgroups. For example, Asian and Pacific Islander individuals were grouped together, despite being from distinct and diverse backgrounds.^55,56^ Similarly, the Hispanic category encompassed individuals of any racial group with varying countries of origin and language preferences.^57^ Third, the latest data examined in this study are from 2019. Although more recent data are available from CMS, the newer data overlap with the COVID-19 pandemic, which disrupted health care use patterns in the US. Fourth, given the limitations of CMS administrative data, we relied on proxies for socioeconomic status and access to care, which may not accurately represent individual differences. Additionally, we did not control for clinician bias in prescribing patterns. Fifth, selecting MA contracts with highly complete data may bias findings toward lower PIM use in MA plans. Last, although the HEDIS algorithms are validated standards, they present several issues. Prescription drug claims may overestimate PIM exposure for patients who fill prescriptions but do not take them. The HEDIS algorithms do not account for individual differences in the number of prescription drugs filled and do not differentiate between PIM initiation and prevalent use. Additionally, the algorithms aggregate multiple drug classes into one measure, which may obscure important differences between racial and ethnic groups in PIM use by drug or by drug class.

## Conclusion

In this cross-sectional study of 21 193 170 Medicare beneficiaries with 32 199 587 beneficiary-year observations from 2016 to 2019, White Medicare beneficiaries had consistently higher rates of PIM use compared with other racial and ethnic groups. Given these findings, CMS should strategically focus health equity initiatives to reduce PIM use among all racial and ethnic groups.

## Notes

Supplement 1. **eMethods.** High-Risk Medication Use and Drug-Disease Interactions **eFigure**. Sample Flow Diagram **eTable 1.** Characteristics of Sample Eligible for Measure of Potentially Harmful Drug-Disease Interactions in Older Adults With Dementia **eTable 2.** Characteristics of Sample Eligible for Measure of Potentially Harmful Drug-Disease Interactions in Older Adults With a History of Falls

Supplement 2.
Data Sharing Statement

## References

[1] 2023 American Geriatrics Society Beers Criteria Update Expert Panel. American Geriatrics Society 2023 updated AGS Beers criteria for potentially inappropriate medication use in older adults. J Am Geriatr Soc. 2023;71(7):2052-2081. doi:10.1111/jgs.18372 37139824 PMC12478568

[2] National Committee for Quality Assurance. Use of high-risk medications in older adults (DAE). Accessed February 28, 2025. https://www.ncqa.org/report-cards/health-plans/state-of-health-care-quality-report/measures-list/use-of-high-risk-medications-in-older-adults-dae

[3] National Committee for Quality Assurance. Potentially harmful drug-disease interactions in older adults (DDE). Accessed February 28, 2025. https://www.ncqa.org/report-cards/health-plans/state-of-health-care-quality-report/measures-list/potentially-harmful-drug-disease-interactions-in-older-adults-dde

[4] Riester MR, Goyal P, Steinman MA, . Prevalence of potentially inappropriate medication prescribing in US nursing homes, 2013-2017. J Gen Intern Med. 2023;38(6):1563-1566. doi:10.1007/s11606-022-07825-6 36175759 PMC10160255

[5] Chang CM, Liu PYY, Yang YHK, Yang YC, Wu CF, Lu FH. Use of the Beers criteria to predict adverse drug reactions among first-visit elderly outpatients. Pharmacotherapy. 2005;25(6):831-838. doi:10.1592/phco.2005.25.6.831 15927902

[6] Freeland KN, Thompson AN, Zhao Y, Leal JE, Mauldin PD, Moran WP. Medication use and associated risk of falling in a geriatric outpatient population. Ann Pharmacother. 2012;46(9):1188-1192. doi:10.1345/aph.1Q689 22872750

[7] Clark CM, Shaver AL, Aurelio LA, . Potentially inappropriate medications are associated with increased healthcare utilization and costs. J Am Geriatr Soc. 2020;68(11):2542-2550. doi:10.1111/jgs.16743 32757494 PMC8235922

[8] Hyttinen V, Jyrkkä J, Valtonen H. A systematic review of the impact of potentially inappropriate medication on health care utilization and costs among older adults. Med Care. 2016;54(10):950-964. doi:10.1097/MLR.0000000000000587 27367864

[9] Schiavo G, Forgerini M, Lucchetta RC, Silva GO, Mastroianni PDC. Cost of adverse drug events related to potentially inappropriate medication use: a systematic review. J Am Pharm Assoc (2003). 2022;62(5):1463-1476.e14. doi:10.1016/j.japh.2022.04.008 35513979

[10] Jungo KT, Choudhry NK, Chaitoff A, Lauffenburger JC. Associations between sex, race/ethnicity, and age and the initiation of chronic high-risk medication in US older adults. J Am Geriatr Soc. 2024;72(12):3705-3718. doi:10.1111/jgs.19173 39215549 PMC11637294

[11] Figueroa JF, Dai D, Feyman Y, . Use of high-risk medications among older adults enrolled in Medicare Advantage plans vs traditional Medicare. JAMA Netw Open. 2023;6(6):e2320583. doi:10.1001/jamanetworkopen.2023.20583 37368399 PMC10300714

[12] Shaver AL, Clark CM, Hejna M, Feuerstein S, Wahler RG Jr, Jacobs DM. Trends in fall-related mortality and fall risk increasing drugs among older individuals in the United States, 1999-2017. Pharmacoepidemiol Drug Saf. 2021;30(8):1049-1056. doi:10.1002/pds.5201 33534172 PMC8254780

[13] Zhu CW, Choi J, Hung W, Sano M. Racial and ethnic disparities in potentially inappropriate medication use in patients with dementia. J Am Geriatr Soc. 2024;72(11):3360-3373. doi:10.1111/jgs.19152 39166851 PMC11560670

[14] Centers for Medicare & Medicaid Services. CMS framework for health equity 2022-2032. 2022. Accessed August 28, 2024. https://www.cms.gov/files/document/cms-framework-health-equity-2022.pdf

[15] Freed M, Fuglesten Biniek J, Damico A, Neuman T. Medicare Advantage in 2024: enrollment update and key trends. Kaiser Family Foundation. August 8, 2024. Accessed September 18, 2024. https://www.kff.org/medicare/issue-brief/medicare-advantage-in-2024-enrollment-update-and-key-trends/

[16] Nothelle SK, Sharma R, Oakes A, Jackson M, Segal JB. Factors associated with potentially inappropriate medication use in community-dwelling older adults in the United States: a systematic review. Int J Pharm Pract. 2019;27(5):408-423. doi:10.1111/ijpp.12541 30964225 PMC7938818

[17] Ochieng N, Fuglesten Biniek J, Cubanski J, Neuman T. Disparities in health measures by race and ethnicity among beneficiaries in Medicare Advantage: a review of the literature. Kaiser Family Foundation. December 13, 2023. Accessed May 31, 2024. https://www.kff.org/report-section/disparities-in-health-measures-by-race-and-ethnicity-among-beneficiaries-in-medicare-advantage-appendix/

[18] Centers for Medicare & Medicaid Services. 2024 Medicare Advantage and Part D Final Rule (CMS-4201-F). April 5, 2023. Accessed June 26, 2024. https://www.cms.gov/newsroom/fact-sheets/2024-medicare-advantage-and-part-d-final-rule-cms-4201-f

[19] Jung J, Carlin C, Feldman R. Measuring resource use in Medicare Advantage using encounter data. Health Serv Res. 2022;57(1):172-181. doi:10.1111/1475-6773.13879 34510453 PMC8763275

[20] Jung J, Carlin C, Feldman R, Tran L. Implementation of resource use measures in Medicare Advantage. Health Serv Res. 2022;57(4):957-962. doi:10.1111/1475-6773.13970 35411550 PMC10501335

[21] Nguyen KH, Oh EG, Meyers DJ, Kim D, Mehrotra R, Trivedi AN. Medicare Advantage enrollment among beneficiaries with end-stage renal disease in the first year of the 21st Century Cures Act. JAMA. 2023;329(10):810-818. doi:10.1001/jama.2023.1426 36917063 PMC10015314

[22] Research Data Assistance Center. Research Triangle Institute (RTI) race code. Accessed July 27, 2021. https://resdac.org/cms-data/variables/research-triangle-institute-rti-race-code

[23] Eicheldinger C, Bonito A. More accurate racial and ethnic codes for Medicare administrative data. Health Care Financ Rev. 2008;29(3):27-42. 18567241 PMC4195038

[24] National Committee for Quality Assurance. HEDIS 2020 Volume 2: Technical Specifications for Health Plans. National Committee for Quality Assurance; 2020.

[25] Alzheimer’s Association. 2023 Alzheimer’s disease facts and figures. Alzheimers Dement. 2023;19(4):1598-1695. doi:10.1002/alz.13016 36918389

[26] Adeyemi A, Delhougne G. Incidence and economic burden of intertrochanteric fracture: a Medicare claims database analysis. JB JS Open Access. 2019;4(1):e0045. doi:10.2106/JBJS.OA.18.00045 31161153 PMC6510469

[27] 2019 American Geriatrics Society Beers Criteria Update Expert Panel. American Geriatrics Society 2019 Updated AGS Beers criteria for potentially inappropriate medication use in older adults. J Am Geriatr Soc. 2019;67(4):674-694. doi:10.1111/jgs.15767 30693946

[28] Weesie J. Seemingly unrelated estimation and the cluster-adjusted sandwich estimator. Stata Tech Bull. 2000;9(52):34-47.

[29] Kim DH, Schneeweiss S, Glynn RJ, Lipsitz LA, Rockwood K, Avorn J. Measuring frailty in Medicare data: development and validation of a claims-based frailty index. J Gerontol A Biol Sci Med Sci. 2018;73(7):980-987. doi:10.1093/gerona/glx229 29244057 PMC6001883

[30] Jung J, Feldman R, Carlin C. Coding intensity through health risk assessments and chart reviews in Medicare Advantage: does it explain resource use? Med Care Res Rev. 2023;80(6):641-647. doi:10.1177/10775587231191169 37542373 PMC11974351

[31] Lim D, Jung J. Racial-ethnic variations in potentially inappropriate psychotropic medication use among the elderly. J Racial Ethn Health Disparities. 2019;6(2):436-445. doi:10.1007/s40615-018-00541-0 30446988

[32] Alvarez PA, Gao Y, Girotra S, Mentias A, Briasoulis A, Vaughan Sarrazin MS. Potentially harmful drug prescription in elderly patients with heart failure with reduced ejection fraction. ESC Heart Fail. 2020;7(4):1862-1871. doi:10.1002/ehf2.12752 32419388 PMC7373931

[33] Jungo KT, Streit S, Lauffenburger JC. Utilization and spending on potentially inappropriate medications by US older adults with multiple chronic conditions using multiple medications. Arch Gerontol Geriatr. 2021;93:104326. doi:10.1016/j.archger.2020.104326 33516154

[34] Klein E, Saheed M, Irvin N, . Racial and socioeconomic disparities evident in inappropriate antibiotic prescribing in the emergency department. Ann Emerg Med. 2024;84(2):101-110. doi:10.1016/j.annemergmed.2023.12.003 38260931

[35] Abraham DS, Pham Nguyen TP, Hennessy S, . Frequency of and risk factors for potentially inappropriate medication use in Parkinson’s disease. Age Ageing. 2020;49(5):786-792. doi:10.1093/ageing/afaa033 32255485 PMC7444670

[36] Martin C, Hales C, Gu Q, Ogden C. Prescription drug use in the United States, 2015-2016. National Center for Health Statistics. May 2019. Accessed September 18, 2024. https://www.cdc.gov/nchs/products/databriefs/db334.htm

[37] Morden NE, Chyn D, Wood A, Meara E. Racial inequality in prescription opioid receipt—role of individual health systems. N Engl J Med. 2021;385(4):342-351. doi:10.1056/NEJMsa2034159 34289277 PMC8402927

[38] Jacobs JA, Addo DK, Zheutlin AR, . Prevalence of statin use for primary prevention of atherosclerotic cardiovascular disease by race, ethnicity, and 10-year disease risk in the US: National Health and Nutrition Examination Surveys, 2013 to March 2020. JAMA Cardiol. 2023;8(5):443-452. doi:10.1001/jamacardio.2023.0228 36947031 PMC10034667

[39] Ilonze O, Free K, Breathett K. Unequitable heart failure therapy for Black, Hispanic and American-Indian patients. Card Fail Rev. 2022;8:e25. doi:10.15420/cfr.2022.02 35865458 PMC9295006

[40] Qato DM, Wilder J, Zenk S, Davis A, Makelarski J, Lindau ST. Pharmacy accessibility and cost-related underuse of prescription medications in low-income Black and Hispanic urban communities. J Am Pharm Assoc (2003). 2017;57(2):162-169.e1. doi:10.1016/j.japh.2016.12.065 28153704 PMC5348265

[41] Agency for Healthcare Research and Quality. 2022 national healthcare quality and disparities report. Updated July 2023. Accessed January 15, 2025. https://www.ahrq.gov/research/findings/nhqrdr/nhqdr22/index.html 38377267

[42] Landon BE, Onnela JP, Meneades L, O’Malley AJ, Keating NL. Assessment of racial disparities in primary care physician specialty referrals. JAMA Netw Open. 2021;4(1):e2029238. doi:10.1001/jamanetworkopen.2020.29238 33492373 PMC7835717

[43] Xu WY, Shooshtari A, Jung J. Disparities in cost-related drug nonadherence under the Affordable Care Act. J Pharm Health Serv Res. 2019;10(2):177-185. doi:10.1111/jphs.12295

[44] Kaplan CM, Waters TM, Clear ER, Graves EE, Henderson S. The impact of prescription drug coverage on disparities in adherence and medication use: a systematic review. Med Care Res Rev. 2024;81(2):87-95. doi:10.1177/10775587231218050 38174355

[45] Hall WJ, Chapman MV, Lee KM, . Implicit racial/ethnic bias among health care professionals and its influence on health care outcomes: a systematic review. Am J Public Health. 2015;105(12):e60-e76. doi:10.2105/AJPH.2015.302903 26469668 PMC4638275

[46] Adams C, Chatterjee A, Harder BM, Mathias LH. Beyond unequal access: acculturation, race, and resistance to pharmaceuticalization in the United States. SSM Popul Health. 2018;4:350-357. doi:10.1016/j.ssmph.2018.04.003 29854920 PMC5976842

[47] Ramsay C, Jacobson G, Findley S, Cicchiello A. Medicare Advantage: a policy primer. January 31, 2024. Accessed March 25, 2025. https://www.commonwealthfund.org/publications/explainer/2024/jan/medicare-advantage-policy-primer

[48] Timbie JW, Bogart A, Damberg CL, . Medicare Advantage and fee-for-service performance on clinical quality and patient experience measures: comparisons from three large states. Health Serv Res. 2017;52(6):2038-2060. doi:10.1111/1475-6773.12787 29130269 PMC5682140

[49] Sen AP, Meiselbach MK, Anderson KE, Miller BJ, Polsky D. Physician network breadth and plan quality ratings in Medicare Advantage. JAMA Health Forum. 2021;2(7):e211816. doi:10.1001/jamahealthforum.2021.1816 35977214 PMC8796886

[50] Tsang CCS, Garuccio J, Dong X, Sim Y, Wang J. Effects of star ratings bonus payments on disparities in medication utilization issues. Explor Res Clin Soc Pharm. 2023;11:100323. doi:10.1016/j.rcsop.2023.100323 37694164 PMC10485150

[51] Freed M, Fuglesten Biniek J, Damico A, Neuman T. Medicare Advantage in 2024: premiums, out-of-pocket limits, supplemental benefits, and prior authorization. Kaiser Family Foundation. August 8, 2024. Accessed November 15, 2024. https://www.kff.org/medicare/issue-brief/medicare-advantage-in-2024-premiums-out-of-pocket-limits-supplemental-benefits-and-prior-authorization/

[52] Aggarwal R, Gondi S, Wadhera RK. Comparison of Medicare Advantage vs traditional Medicare for health care access, affordability, and use of preventive services among adults with low income. JAMA Netw Open. 2022;5(6):e2215227. doi:10.1001/jamanetworkopen.2022.15227 35671058 PMC9175080

[53] Gangopadhyaya A, Zuckerman S, Rao N. Assessing the difference in racial and ethnic disparities in access to and use of care between traditional Medicare and Medicare Advantage. Health Serv Res. 2023;58(4):914-923. doi:10.1111/1475-6773.14150 36894493 PMC10315374

[54] Kruse G, Lopez-Carmen VA, Jensen A, Hardie L, Sequist TD. The Indian Health Service and American Indian/Alaska Native health outcomes. Annu Rev Public Health. 2022;43:559-576. doi:10.1146/annurev-publhealth-052620-103633 35081315

[55] Dhanjani SA, Yang HH, Goyal S, Zhang K, Gee G, Cowgill B. Trends in health care access disparities among Asian and Pacific Islander health fair participants in Los Angeles, 2011-2019. Public Health Rep. 2023;138(1):97-106. doi:10.1177/00333549211061328 35067110 PMC9730186

[56] Bang SH, Huang YC, Kuo HJ, Cho ES, García AA. Health status and healthcare access of Southeast Asian refugees in the United States: an integrative review. Public Health Nurs. 2023;40(2):324-337. doi:10.1111/phn.13167 36662767

[57] Aragones A, Hayes SL, Chen MH, González J, Gany FM. Characterization of the Hispanic or Latino population in health research: a systematic review. J Immigr Minor Health. 2014;16(3):429-439. doi:10.1007/s10903-013-9773-0 23315046 PMC4518558

