# Supplementary material for: Racial and Ethnic Differences in Potentially Inappropriate Medication Use Among Medicare Beneficiaries
Source: JAMA Netw Open. 2025 Apr 14;8(4):e254763. doi: 10.1001/jamanetworkopen.2025.4763 (PMC11997722; doi:10.1001/jamanetworkopen.2025.4763)
Supplement: Supplement 1. — eMethods. High-Risk Medication Use and Drug-Disease Interactions eFigure. Sample Flow Diagram eTable 1. Characteristics of Sample Eligible for Measure of Potentially Harmful Drug-Disease Interactions in Older Adults With Dementia eTable 2. Characteristics of Sample Eligible for Measure of Potentially Harmful Drug-Disease Interactions in Older Adults With a History of Falls [file jamanetwopen-e254763-s001.pdf]

Supplementary Online Content

Raver E, Jung J, Carlin C, Feldman R, Retchin S, Xu W. Racial and ethnic differences in potentially inappropriate medication use by Medicare beneficiaries. *JAMA Netw Open.* 2025;8(4):e254763. doi:10.1001/jamanetworkopen.2025.4763

eMethods. High-Risk Medication Use and Drug-Disease Interactions

eFigure. Sample Flow Diagram

eTable 1. Characteristics of Sample Eligible for Measure of Potentially Harmful Drug-Disease Interactions in Older Adults With Dementia

eTable 2. Characteristics of Sample Eligible for Measure of Potentially Harmful Drug-Disease Interactions in Older Adults With a History of Falls

This supplementary material has been provided by the authors to give readers additional information about their work.

© 2025 Raver E et al. *JAMA Network Open*.

eMethods. High-Risk Medication Use and Drug-Disease Interactions

High-risk medication use in older adults

This outcome measures the proportion of Medicare beneficiaries who were dispensed the same high-risk medication at least twice during the calendar year. High-risk medications include anticholinergics (first-generation antihistamines, anti-Parkinson agents), antispasmodics, dipyridamole, tricyclic antidepressants, centrally-acting alpha agonists, disopyramide, immediate release nifedipine, barbiturates, ergoloid mesylates, isoxsuprine, meprobomate, estrogens, long-acting sulfonylureas, indomethacin, ketorolac, meperidine, and skeletal muscle relaxants. Additionally, nitrofurantoin and non-benzodiazepine hypnotics are considered high-risk medications if dispensed for more than a 90 days' supply. Similarly, reserpine, digoxin, and doxepin are considered high risk medications if the average daily dose exceeds specified thresholds.

Potentially harmful drug-disease interactions in older adults with dementia

This outcome measures the proportion of Medicare beneficiaries with ADRD who filled a prescription for a potentially harmful medication. Potentially harmful medications for this outcome include antipsychotics, benzodiazepines, non-benzodiazepine hypnotics, tricyclic antidepressants, and anticholinergic agents (antiemetics, first-generation antihistamines, antispasmodics, oral antimuscarinics, anti-Parkinson agents, and skeletal muscle relaxants). Beneficiaries with diagnoses of psychosis, schizophrenia, schizoaffective disorder, or bipolar disorder were excluded from this outcome.

Potentially harmful drug-disease interactions in older adults who had an accidental fall or hip fracture

This outcome measures the proportion of Medicare beneficiaries with ADRD who had an accidental fall or hip fracture and who subsequently filled a prescription for a potentially harmful medication. Accidental falls and fractures were identified by inpatient, outpatient, observation, or emergency department visits associated with a diagnosis of accidental falls or hip fracture. Potentially harmful medications for this outcome include anticonvulsants, SSRIs, SNRIs, antipsychotics, benzodiazepines, non-benzodiazepine hypnotics, and tricyclic antidepressants. Beneficiaries with diagnoses of psychosis, schizophrenia, schizoaffective disorder, bipolar disorder, major depressive disorder, or seizure disorder were excluded from this outcome.

For each outcome measure, potentially inappropriate medication use included all medications in the drug classes listed above. The precise lists of medications are available in the Healthcare Effectiveness Data and Information Set (HEDIS) Medication List Directory. The list of ICD-10 diagnosis codes associated with conditions is available in the HEDIS Value Set Directory. HEDIS measure specifications are proprietary information and may only be used pursuant to a licensing agreement with the National Committee for Quality Assurance.

eFigure. Sample Flow Diagram

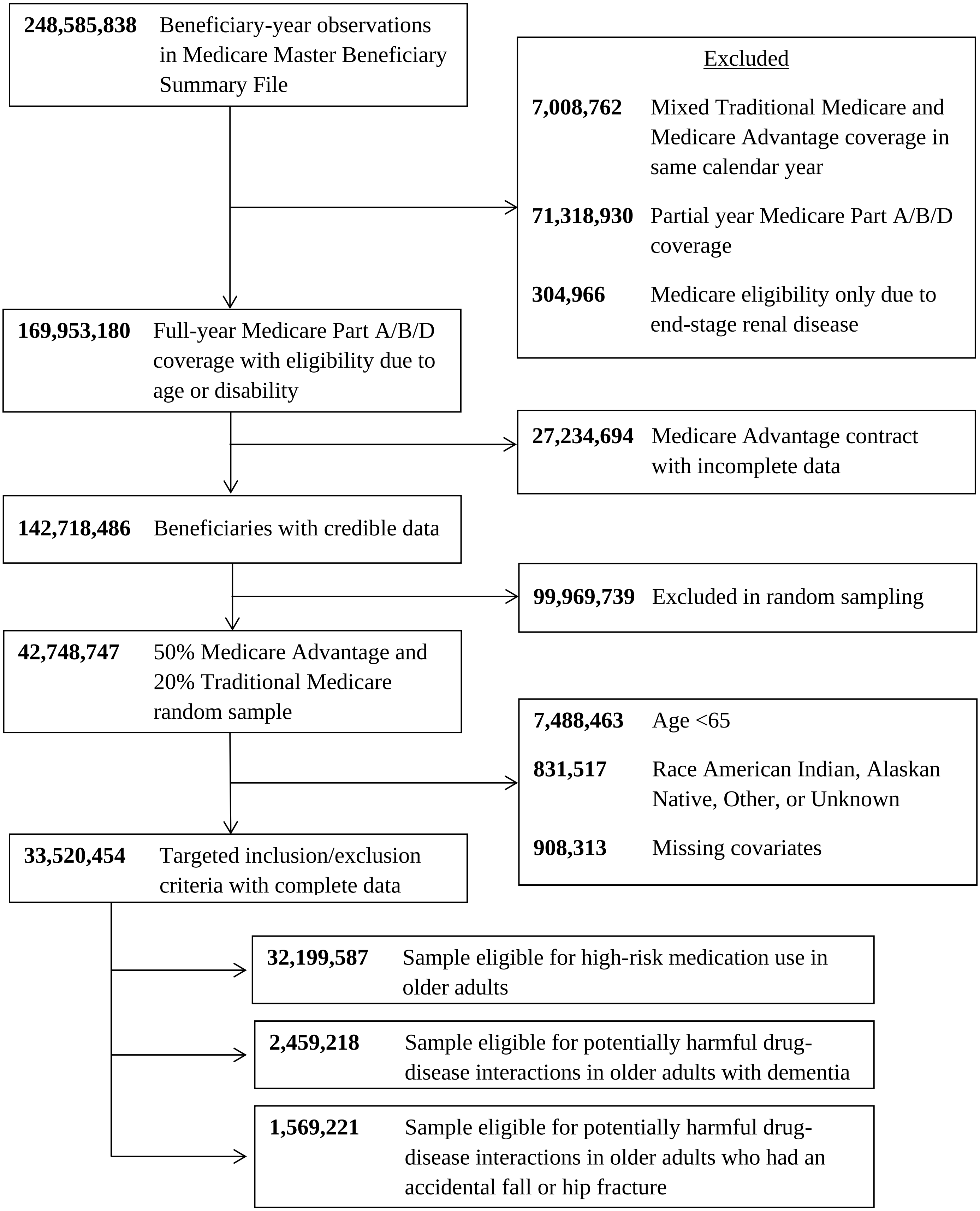

© 2025 Raver E et al. *JAMA Network Open*.

eTable 1. Characteristics of Sample Eligible for Measure of Potentially Harmful Drug-Disease Interactions in Older Adults With Dementia

Characteristic	Overall	White	Black	Asian or Pacific Islander	Hispanic
Number of patient-year observations[a]	2,459,218	1,833,657	289,232	72,835	263,494
Number of patients	1,856,144	1,390,433	214,842	57,031	193,838
Age, mean (SD)	82.7 (7.7)	83.0 (7.6)	81.5 (7.8)	82.5 (7.8)	81.5 (7.6)
Female, n (%)	1,586,327 (64.5)	1,176,956 (64.2)	195,648 (67.6)	45,441 (62.4)	168,282 (63.9)
Male, n (%)	872,891 (35.5)	656,701 (35.8)	93,584 (32.4)	27,394 (37.6)	95,212 (36.1)
Rural residence, n (%)	477,203 (19.4)	410,492 (22.4)	42,965 (14.9)	2562 (3.5)	21,184 (8.0)
Dual-eligible, n (%)	732,309 (29.8)	394,990 (21.5)	135,178 (46.7)	44,781 (61.5)	157,360 (59.7)
Medicare Advantage enrollment, n (%)	1,405,612 (57.2)	993,511 (54.2)	194,125 (67.1)	38,751 (53.2)	179,225 (68.0)
HCC risk score, mean (SD)	1.73 (1.31)	1.68 (1.25)	1.92 (1.54)	1.65 (1.27)	1.88 (1.38)
Kim frailty score, mean (SD)	0.25 (0.08)	0.25 (0.08)	0.25 (0.09)	0.22 (0.08)	0.24 (0.08)
ZIP code median household income, mean (SD)	64343 (25909)	67053 (25798)	51174 (21562)	77408 (30458)	56329 (22678)
Percentage of ZIP code with a 4-year degree, mean (SD)	30.5 (16.4)	31.9 (16.5)	24.9 (14.4)	37.5 (17.2)	25.1 (14.5)
Percentage of ZIP code households speaking only English, mean (SD)	79.8 (21.2)	85.0 (15.0)	82.6 (17.3)	58.0 (22.5)	46.5 (26.9)
Hospital beds per 1000 population (county level), mean (SD)	3.05 (2.05)	2.97 (2.07)	3.68 (2.43)	2.78 (1.32)	3.02 (1.32)
Physicians per 1000 population (county level), mean (SD)	3.34 (2.27)	3.17 (2.20)	3.92 (2.54)	4.39 (2.40)	3.62 (2.20)

Abbreviation: HCC, hierarchical condition category.

[a] Percentages are calculated using the number of patient-year observations as the denominator.

© 2025 Raver E et al. *JAMA Network Open*.

eTable 2. Characteristics of Sample Eligible for Measure of Potentially Harmful Drug-Disease Interactions in Older Adults With a History of Falls

Characteristic	Overall	White	Black	Asian or Pacific Islander	Hispanic
Number of patient-year observations[a]	1,569,221	1,287,386	129,059	36,432	116,344
Number of patients	1,347,835	1,106,660	110,798	31,748	98,629
Age, mean (SD)	80.4 (8.13)	80.7 (8.1)	78.6 (8.1)	80.4 (8.3)	79.2 (7.9)
Female, n (%)	1,044,256 (66.5)	850,942 (66.1)	91,752 (71.1)	24,475 (67.2)	77,087 (66.3)
Male, n (%)	524,965 (33.5)	436,444 (33.9)	37,307 (28.9)	11,957 (32.8)	39,257 (33.7)
Rural residence, n (%)	331,618 (21.1)	298,794 (23.2)	18,674 (14.5)	1612 (4.4)	12,538 (10.8)
Dual-eligible, n (%)	261,675 (16.7)	143,700 (11.2)	49,223 (38.1)	15,798 (43.4)	52,954 (45.5)
Medicare Advantage enrollment, n (%)	893,613 (56.9)	699,210 (54.3)	91,625 (71.0)	20,785 (57.1)	81,993 (70.5)
HCC risk score, mean (SD)	1.48 (1.15)	1.45 (1.12)	1.67 (1.33)	1.46 (1.16)	1.58 (1.22)
Kim frailty score, mean (SD)	0.20 (0.07)	0.20 (0.07)	0.21 (0.08)	0.19 (0.07)	0.20 (0.07)
ZIP code median household income, mean (SD)	65697 (26106)	67560 (25870)	50482 (21474)	80374 (31410)	57366 (23121)
Percentage of ZIP code with a 4-year degree, mean (SD)	31.3 (16.7)	32.3 (16.6)	25.0 (14.5)	39.7 (17.8)	25.1 (15.1)
Percentage of ZIP code households speaking only English, mean (SD)	82.3 (18.5)	85.5 (14.3)	83.0 (17.2)	63.2 (21.4)	51.1 (26.3)
Hospital beds per 1000 population (county level), mean (SD)	3.04 (2.09)	2.97 (2.10)	3.78 (2.49)	2.87 (1.39)	2.98 (1.40)
Physicians per 1000 population (county level), mean (SD)	3.34 (2.36)	3.21 (2.30)	4.12 (2.72)	4.49 (2.51)	3.54 (2.27)

Abbreviation: HCC, hierarchical condition category.

[a] Percentages are calculated using the number of patient-year observations as the denominator.

© 2025 Raver E et al. *JAMA Network Open*.
