# Supplementary material for: Racial and Ethnic Differences in Potentially Inappropriate Medication Use Among Medicare Beneficiaries
Source: JAMA Netw Open. 2025 Apr 14;8(4):e254763. doi: 10.1001/jamanetworkopen.2025.4763 (PMC11997722; doi:10.1001/jamanetworkopen.2025.4763)
Supplement: Supplement 2. — Data Sharing Statement [file jamanetwopen-e254763-s002.pdf]

Data Sharing Statement

Raver. Racial and Ethnic Differences in Potentially Inappropriate Medication Use Among Medicare Beneficiaries. *JAMA Netw Open*. Published April 14, 2025.
doi:10.1001/jamanetworkopen.2025.4763

Data
Data available: No

Additional Information
Explanation for why data not available: The data used in this study contain protected health information and are subject to a data use agreement with the Centers for Medicare & Medicaid Services, which restricts sharing the data. All our aggregated statistical results, including those unreported, are available to the public upon request to the corresponding author, Wendy Y. Xu.
